# Peer Support for Women with Heart Disease: Program Description and Evaluation of Women@Heart

**DOI:** 10.1016/j.cjco.2025.08.008

**Published:** 2025-08-25

**Authors:** Nadine Elias, Dan Yedu Quansah, Lisa A. McDonnell, Thais Coutinho, Robert D. Reid, Hassan Mir, Kerri-Anne Mullen

**Affiliations:** a Canadian Women’s Heart Health Centre, University of Ottawa Heart Institute, Ottawa, Ontario, Canada; b Division of Cardiac Prevention and Rehabilitation, University of Ottawa Heart Institute, Ottawa, Ontario, Canada; c School of Epidemiology and Public Health, Faculty of Medicine, University of Ottawa, Ottawa, Ontario, Canada; d Champlain Regional Stroke Network, The Ottawa Hospital, Ottawa, Ontario, Canada; e Department of Cardiovascular Medicine, Mayo Clinic, Rochester, Minnesota, USA; f Division of Cardiology, Department of Medicine, University of Ottawa Heart Institute, University of Ottawa, Ottawa, Ontario, Canada

**Keywords:** peer support, Women@Heart, cardiovascular disease, women, psychosocial, health behavior, feasibility

## Abstract

Women with heart disease face higher risks of death, cardiac events, depression, and reduced quality of life, compared to men. However, women’s participation level in cardiac rehabilitation programs remains low. Peer support programs, such as Women@Heart (W@H), led by women with lived experiences, can enhance program uptake and improve psychosocial well-being. Our pilot evaluation of W@H showed improvements in psychosocial and health behaviour measures, including coping skills and health empowerment. This program evaluation demonstrates the feasibility and effectiveness of W@H in addressing the unique needs of women with heart disease; however, further and large-scale evaluation is needed.

## Résumé

Les femmes atteintes de cardiopathies courent un risque plus élevé de décès, d'événements cardiaques, de dépression et de diminution de leur qualité de vie que les hommes. Cependant, la participation des femmes aux programmes de réadaptation cardiaque reste faible. Les programmes de soutien par les pairs, tels que Women@Heart (W@H), dirigés par des femmes ayant vécu cette expérience, peuvent améliorer l'adhésion au programme et le bien-être psychosocial. Notre évaluation pilote du programme W@H a mis en évidence des améliorations des paramètres psychosociaux et des comportements liés à la santé, notamment les capacités d'adaptation et d'autonomisation en matière de santé. Cette évaluation démontre la faisabilité et l'efficacité du programme W@H pour répondre aux besoins spécifiques des femmes atteintes d'une maladie cardiaque; toutefois, une évaluation plus approfondie et à plus grande échelle est nécessaire.

Women with cardiovascular disease (CVD) face disproportionately poorer outcomes, compared to men.^1^ They also are more likely to report depression and a lower quality of life following a cardiovascular diagnosis.^1^^,^^2^ Women indicate a greater need to talk about their experiences with CVD and to receive social support to help them cope.^3^ Despite the fact that cardiac rehabilitation (CR) programs have been designed to address these issues, women are 30% less likely than men to participate in CR, citing barriers such as lack of emotional support, gendered communication styles, caregiving responsibilities, and geographic or logistic challenges.^4^^,^^5^

This underutilization highlights a critical care gap. Although peer-support interventions (assistance from others who share a common experience) have demonstrated benefits in enhancing recovery support for women with CVD, self-care behaviours, and psychosocial well-being,^5^, ^6^, ^7^, ^8^ their implementation and evaluation specifically among women remain limited. Few studies have examined gender-specific peer-support models, and existing evidence rarely disaggregates outcomes by sex or gender. Previous gender-specific analyses indicate that social support positively influences health behaviours, promoting smoking cessation, increased physical activity, healthier eating habits, reduced alcohol intake, and better adherence to medical treatments.^9^ Peer support, has been found to positively affect psychological and physical health outcomes by contributing to a patient’s sense of validation, normalizing the illness experience, reducing social and emotional isolation, and enhancing knowledge and self-efficacy.^9^ Peer support can influence an individual’s response to stressors, through information sharing and the modelling of effective coping strategies.^9^ A systematic review of randomized controlled trials (RCTs) demonstrated positive effects of peer-support interventions among male and female patients with heart disease, including increased self-efficacy, improved physical activity, reduced pain, and fewer emergency room visits.^10^

Women with CVD have identified peer support from other women as essential to their recovery following a cardiac event^8^; however, as the evidence is sparse, little is known about the effectiveness or scalability of peer-led interventions tailored to women recovering from heart disease. A clear need is the evaluation of peer-support interventions that are specifically designed to meet the unique needs of women with CVD. To address this gap, the Canadian Women’s Heart Health Centre at the University of Ottawa Heart Institute (UOHI) developed Women@Heart (W@H), a community-based peer-support program led by women with lived experience of CVD. The program was designed to meet the unique psychosocial and informational needs of women, with the goal of complementing traditional care and improving recovery experiences. This article presents program evaluation findings from the W@H program, conducted prior to the launch of a formal comparative study (NCT03286010). The objectives of this evaluation were as follows: (i) to assess the feasibility of delivering the W@H program; and (ii) to estimate the effects of the W@H program on psychosocial outcomes and health behaviours.

## Methods

### Design and recruitment

A before-and-after, repeated-measures design was used. Prior to the COVID-19 pandemic, the W@H program was delivered entirely in person, to groups within community settings. During the pandemic, the program was adapted for virtual delivery via videoconferencing. This evaluation focuses specifically on the pre-pandemic in-person W@H format. All female patients discharged from the UOHI between 2015 and 2018 were prescreened to determine eligibility for the W@H program, based on location (lived within a ≤ 30-minute drive of the city of Ottawa, Ontario, Canada) and language spoken (English or French). All potentially eligible patients were called to provide information about the W@H program, and, if they were interested, patients were enrolled in an upcoming group. This protocol was reviewed and approved by the Ottawa Health Sciences Network Research Ethics Board as a quality-improvement evaluation. This evaluation was intended to assess the feasibility of program delivery and explore potential changes in psychosocial and behavioural measures to inform the design of the larger, formal Women@Heart study (NCT03286010), which is currently ongoing to evaluate the program’s long-term impact.

### The W@H program

The W@H peer-support program emphasized emotional and social support by fostering mutual understanding and empathy, and reducing isolation through shared experiences. The program promoted empowerment by encouraging participants to share their stories, reflect on their journeys, and build confidence through mutual encouragement. The program was person-centred, adapting to individual emotional needs and each participant’s personal pace of recovery. In addition, the program cultivated a sense of belonging, helping to foster a sense of community and sustain long-term relationships beyond the duration of the program. Compared to traditional CR programs, which are typically delivered by healthcare professionals and focus on structured exercise, risk-factor modification, and clinical monitoring, the W@H program is uniquely characterized by group-based facilitation by trained peers with lived experience of heart disease. This model offers emotional validation, shared storytelling, and empowerment that may be particularly resonant with women. Peer support allows participants to express concerns, develop coping strategies, and build community in a less clinical and more relatable setting.

**Figure 1:**
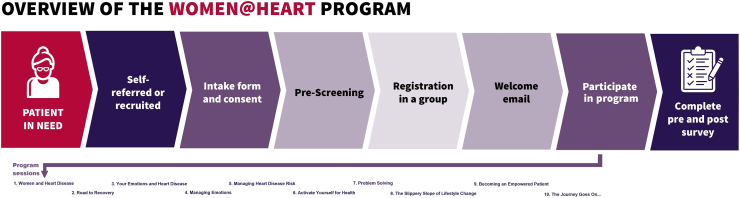
A summary of the Woman@Heart program and curriculum.

The W@H curriculum is evidence-based and was designed by a multidisciplinary committee of program administrators, clinicians, and women with lived experience of CVD, with the following objectives: to enhance coping and reduce risk of isolation among women diagnosed with CVD; to empower women to better understand their condition and take charge of their cardiovascular health; and to build a caring environment for women to learn from and share experiences related to heart health. Figure 1 summarizes the program and curriculum. The in-person W@H group sessions were delivered during 12 two-hour sessions held biweekly over 6 months in a closed group format. The sessions were led by W@H peer leaders—volunteers who had recovered from CVD and felt emotionally and physically well enough to support others in their recovery journey. Peer leaders were selected through a structured screening process assessing their communication skills, reliability, emotional readiness, and interest in group facilitation. All W@H peer leaders were carefully screened based on predetermined criteria to assume a peer-support leadership role. They each completed a 3-day training program focused on the core competencies needed to effectively deliver the intervention, including the standardized intervention curriculum, sequence of the intervention activities, and group facilitation skills.^11^ The intervention sessions focused on the following areas: emotional support (sharing your story, road to recovery, exploration of feelings, coping with changes, emotional management, coping with distress, effective communication, empowerment); informational support (self-care behaviours, risk-factor education and management, healthcare system and community resource navigation); and appraisal support (goal-setting, action-planning, problem-solving, and relapse prevention). The leader-to-participant ratio was 1:6-12. Peer leaders administered sessions codified in a program facilitation manual. Participants received a notebook that reflected the topics of each session. Supplemental Appendix S1 provides an overview and description of the W@H sessions.

### Measures

To assess program feasibility, the following information was gathered: the number of women admitted to UOHI with CVD over the evaluation period; the number of women screened; the number of women enrolled in W@H; and the average number of sessions completed. Feasibility also was assessed using multiple descriptive indicators, including the number of eligible women screened and enrolled, retention and session attendance rates, and participant satisfaction scores. These indicators align with key domains of feasibility studies, including demand, implementation, and acceptability. Although no predefined cutoffs were applied, these measures provided insight into the practicality and acceptability of delivering the W@H program in a real-world setting.

Demographic, psychosocial, and health behaviour information was collected at baseline and at the end of the program (6 months). Psychosocial variables were measured based on their established relevance to cardiovascular risk reduction, their frequent use in CR research, their feasibility in community-based settings, and their known influence on recovery, self-care behaviours, and the program. These variables were as follows: adaptive and maladaptive coping (Brief COPE Inventory); anxiety and depression levels (Hospital Anxiety and Depression Scale); stress (Perceived Stress Scale); and health activation (Patient Activation Measure). Fruit and vegetable consumption (Rapid Eating Assessment for Patients) was assessed as a key dietary behaviour given its strong association with cardiovascular health outcomes and its inclusion in both Canadian and international dietary guidelines. Similarly, moderate-to-vigorous physical activity level (physical activity vital sign) was chosen as a modifiable risk factor targeted by secondary prevention guidelines. Anthropometric measures, including waist circumference, weight, and height, were measured, and body mass index was calculated. Satisfaction with program content, frequency, group dynamic, and leader skill were gathered using an 8-item survey.

### Statistical analyses

Baseline characteristics of participants and feasibility outcomes were summarized using descriptive statistics. One-way repeated measures analysis of variance (RM-ANOVA) was used to assess changes in psychosocial and behavioural outcomes from baseline to end of the program. Results were presented for the total sample as well as by CR participation, stratified by those who participated in W@H only and those who participated in W@H plus CR (W@H+CR). Missing data were observed in 2.5% of cases across key outcome variables. Multiple imputation was performed using 5 imputed datasets, addressing missing values for psychosocial and behavioural outcomes, including coping scores, Hospital Anxiety and Depression Scale, Perceived Stress Scale, Patient Activation Measure, dietary intake, and moderate-to-vigorous physical activity. Imputation was conducted under the assumption that data were missing at random, which was supported by preliminary missingness pattern analysis. Analyses were performed using SPSS version 25 (SPSS, Chicago, IL). All statistical significance was 2-sided and was accepted at *P* < 0.05.

## Results

W@H groups took place in 16 communities around Ottawa, Ontario between January 2015 and March 2019. Thirty-three peer leaders were trained during the evaluation period. In total, 1425 women hospitalized with CVD between 2015 and 2018 were considered eligible for the program, of which 1169 (82.0%) were screened. A total of 482 (41.2%) enrolled in one of 59 W@H groups, and 368 (76.3%) completed the program. Participants completed, on average, 78% of the W@H sessions (> 9 of 12). Of program completers, 309 (84.0%) enrolled in the pre- and post-program evaluations and are included in this analysis.

A total of 98 women (31.7%) participated in W@H only, and 211 (68.3%) participated in W@H+CR. The mean age of participants was 65.6 years (± 9.7). The majority were married or living in a common law partnership (59.9%), were retired (65.0%), and had completed postsecondary education (68.6%). Primary cardiac conditions were as follows: coronary artery disease (67.3%, including 3.2% spontaneous coronary artery dissection); valve disease (12.3%); heart failure (9.4%); arrhythmia (7.8%); and, other (3.2%). The only baseline characteristic that was significantly different between groups was diagnosis—a greater proportion of women in the W@H+CR group had coronary artery disease (68.7%) compared to the number in the W@H-only group (61.1%), and fewer W@H+CR participants had arrhythmia (4.3% vs 17.3%, respectively).

**Figure 2:**
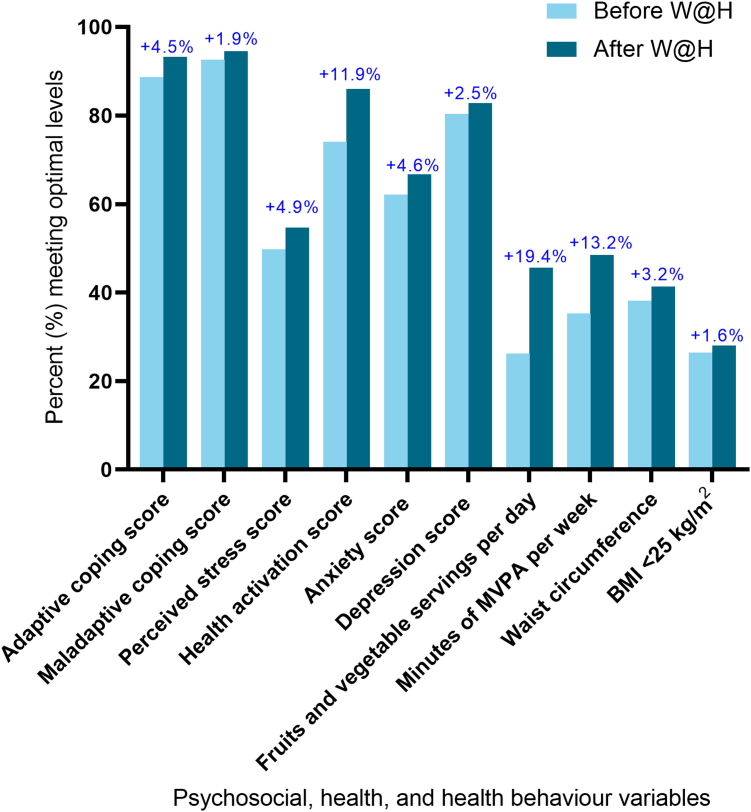
Percentage of participants meeting “normal” or “optimal” guideline levels before and after the Women@Heart (W@H) intervention. For *P*-values associated with these changes, see Table 1. Adaptive coping score indicates % reporting moderate to high use of adaptive coping; maladaptive coping score indicates % reporting low to mild use of maladaptive coping; perceived stress score indicates % reporting low stress; health activation score indicates % taking action or maintaining/pushing further; anxiety score indicates % in normal range; depression score indicates % in normal range; fruits and vegetable servings per day indicates % with ≥ 7 servings; minutes of moderate-to-vigorous physical activity (MVPA) per week indicates % reporting ≥ 150 minutes; waist circumference (cm) indicates % with measurement of < 88 cm. BMI, body mass index.

Psychosocial health and health behaviour outcomes are summarized in Table 1. Statistically significant improvements were observed across multiple outcomes, including adaptive coping (*P* < 0.001), health activation (*P* < 0.001), anxiety (*P* = 0.01), depression (*P* = 0.01), fruit and vegetable intake (*P*< 0.001), and physical activity (*P* < 0.001). However, these pre–post improvements corresponded to small effect sizes (Cohen's d ranging from 0.1 to 0.4). Changes in maladaptive coping, waist circumference, and body mass index were not statistically significant. These findings were consistent across both the W@H-only and the W@H+CR subgroups. Both the W@H-only and the W@H+CR groups had improvements in the proportion of participants who scored within normal or optimal guideline ranges for psychosocial and health behaviour measures (Fig. 2). Average satisfaction with W@H was rated at 4.5 of 5.0. Open-text responses as to what participants liked, suggestions for improvement, and what was considered unhelpful are summarized in the Supplemental Appendix S2.

**Table 1:** Psychosocial, health, and health behaviour variables by group, before and after Women@Heart (W@H) program participation

Variable	Mean (standard deviation)			Cohen’s d	
	Before W@H	After W@H	Δ in mean		*P*
Adaptive coping score					
Total	39.1 (9.0)	41.1 (9.2)	+ 2.0	0.22	< 0.001
W@H only	39.1 (8.5)	41.5 (9.3)	+ 2.4	0.27	< 0.001
W@H with CR	39.2 (9.9)	40.2 (8.8)	+ 1.0	0.11	ns
Maladaptive coping score					
Total	19.7 (5.1)	19.4 (5.2)	– 0.3	– 0.06	ns
W@H only	19.0 (4.8)	18.6 (5.7)	– 0.4	– 0.08	ns
W@H with CR	20.1 (5.2)	19.7 (4.9)	– 0.4	– 0.08	ns
Perceived stress score					
Total	14.3 (7.6)	13.0 (7.5)	– 1.3	– 0.17	< 0.001
W@H only	12.8 (6.7)	11.8 (7.2)	– 1.0	– 0.14	< 0.05
W@H with CR	15.1 (7.9)	13.6 (7.6)	– 1.5	– 0.19	< 0.001
Health activation score					
Total	65.5 (14.6)	68.8 (14.6)	+ 3.3	0.23	< 0.001
W@H only	68.4 (14.3)	71.6 (15.1)	+ 3.2	0.22	< 0.01
W@H with CR	64.2 (14.5)	67.4 (14.2)	+ 3.2	0.22	< 0.001
Anxiety score					
Total	6.6 (4.1)	6.0 (3.9)	– 0.6	– 0.15	< 0.01
W@H only	5.7 (3.4)	5.0 (3.6)	– 0.7	– 0.20	< 0.01
W@H with CR	7.0 (4.3)	6.5 (4.0)	– 0.5	– 0.12	< 0.01
Depression score					
Total	4.6 (3.8)	4.0 (3.4)	– 0.6	– 0.17	< 0.01
W@H only	4.0 (3.5)	3.6 (3.2)	– 0.4	– 0.12	ns
W@H with CR	4.8 (3.9)	4.2 (3.5)	– 0.6	– 0.16	< 0.01
Number of servings of fruits and vegetables per day					
Total	5.7 (1.8)	6.4 (1.9)	+ 0.7	0.38	< 0.001
W@H only	5.8 (1.8)	6.3 (1.8)	+ 0.5	0.28	< 0.01
W@H with CR	5.6 (1.9)	6.4 (2.0)	+ 0.8	0.41	< 0.001
Minutes of moderate-to-vigorous physical activity per week					
Total	139.1 (164.2)	173.7 (159.7)	+ 34.6	0.21	< 0.001
W@H only	134.3 (146.7)	178.7 (163.6)	+ 44.4	0.29	< 0.01
W@H with CR	141.3 (171.9)	171.4 (158.2)	+ 30.1	0.18	< 0.01
Waist circumference, cm					
Total	94.5 (14.5)	93.7 (14.5)	– 0.8	– 0.06	< 0.05
W@H only	92.8 (13.7)	91.6 (13.1)	– 1.2	– 0.09	ns
W@H with CR	95.3 (15.0)	94.7 (15.1)	– 0.6	– 0.04	ns
Body mass index, kg/m^2^					
Total	30.3 (8.0)	29.7 (7.3)	– 0.6	– 0.08	ns
W@H only	28.7 (5.9)	28.4 (5.7)	– 0.3	– 0.05	ns
W@H with CR	31.0 (8.7)	30.4 (7.9)	– 0.6	– 0.07	ns

Total n = 309; W@H with cardiac rehabilitation (CR), n = 211; W@H without CR, n = 98.

ns, not significant.

## Discussion

In this feasibility evaluation of the W@H peer-support program, we found program delivery to be feasible, with high participant satisfaction, and we observed significant improvements in psychosocial and health behaviour outcomes at the end of the program. Results were similar for participants who attended W@H only and participants who attended W@H alongside traditional CR. This similarity suggests that the peer-support intervention may offer unique psychosocial and behavioural benefits that are not attributable to only participation in traditional CR programs. These findings suggest that W@H can be a complementary or standalone model of support for women who may be unable or unwilling to participate in formal CR programs.

Some of our evaluation findings are consistent with previous studies that show that peer-support interventions may be feasible and effective in cardiac populations.^12^ A systematic review with evidence from 12 clinical trials reported improvements in depression, anxiety, quality of life, self-care, and medication adherence following peer-support interventions in mixed-gender samples.^13^ However, a recent review (16 RCTs) found no significant effects on anxiety, depression, health-related quality of life, or perceived social support, despite improvements in self-management and reduction in hospital readmissions.^14^ In contrast, our evaluation found statistically small but meaningful improvements in anxiety and depression scores, as well as adaptive coping and health activation. These differences may reflect the gender-specific design and delivery of W@H, which addressed unique recovery needs of women in a supportive peer environment over an extended intervention period. An important point to note is that previous reviews have not reported sex- or gender-stratified data, thereby limiting direct comparisons and highlighting a critical gap in the evidence base for women-specific interventions.^15^

Further studies are needed to determine if a particular mode is optimal for delivering peer support. W@H involved multiple group sessions conducted over several months and delivered by trained peer leaders, which may enhance the opportunity for recovery, learning, and social support. Additionally, W@H chose frequent and prolonged contact based on available evidence showing the following: frequent contact with individuals helps establish trust between the provider and the individual; scheduled follow-up sessions as a core component of intervention programs are more effective; and prolonged time courses of follow-up facilitate success across sequential stages of behaviour change.^16^ Other formats have shown mixed results, including a telephone-based intervention that improved psychosocial outcomes among men,^15^ and a single 45-minute peer-support group session that led to no desirable effects on stress, depression, relaxation, satisfaction, or pain in patients with heart disease undergoing angiography.^17^ But our findings suggest that longer-duration, structured group formats may be particularly beneficial for women.

The consistent improvements across psychosocial and behavioural outcomes in our study support the idea that repeated, interpersonal engagement in a supportive group context may be key to effectiveness. The evidence suggests that peer-support models delivered in a group-based face-to-face manner such as W@H are more effective in improving perceived social support.^13^^,^^14^ The COVID-19 pandemic led to the transition of the W@H program to virtual groups (videoconference). The evaluation of this virtual model of program delivery has yet to be completed. A 2025 review and meta-analysis evaluating the effects of digital peer-support interventions on general physical health (47 studies) and mental health (73 studies) found a moderate effect for improving physical health and a large effect for improving mental health. The results were similar across age groups and health conditions.^18^ As peer-support programs, including less-formal social media formats, are becoming more common, well-designed studies with long follow-up durations are needed to confirm their impact on health outcomes and psychological well-being. Future studies should explore how factors such as delivery mode (eg, virtual vs in-person), session frequency, and program length influence outcomes across different populations, including women who are less likely to attend traditional CR.

Shared peer education on the necessity of stress management, maintaining physical activity, and adopting coping strategies has been shown to help improve patients’ outcomes.^13^ Shared experiences can create a sense of comfort among peers and patients, allowing patients to communicate their concerns and receive practical solutions in a language that they understand. An important finding to note is that peer-support curricula based on emotional, informational, and appraisal support not only provides patients with useful information in terms of a healthy lifestyle and daily life problem-solving strategies, but also helps them achieve self-efficacy for accomplishing such activities, which ultimately can improve self-care and quality of life.^19^ This evaluation provides preliminary evidence that structured peer support may help women with heart disease progress through recovery and improve their health-related outcomes.

### Strengths and limitations

Our evaluation adds to the evidence regarding the feasibility and effects of peer-support interventions designed and delivered specifically for women recovering from heart disease. Among the strengths of our evaluation is that the program was delivered by trained peer leaders using a standardized curriculum. The evaluation completion rate among participants was high, and several psychosocial, health, and health behaviour factors were measured.

The main limitation was the lack of a control group in this pre-post design, as well as the lack of a CR-only comparator group. However, we were able to compare results between those who participated in the W@H peer-support intervention only and those who participated in both W@H and CR. Data from this pilot were used to inform a matched cohort study, which is currently nearing completion (NCT03286010).

The interpretations of the results from this feasibility evaluation are limited by the lack of sociodemographic data, including ethnicity and socioeconomic status. Additionally, we did not collect demographic or clinical information from women who declined to participate in the program, precluding analysis of potential differences between participants and nonparticipants, which raises a concern of potential selection bias. Although participant satisfaction was assessed, the experience and satisfaction of peer leaders were not formally evaluated. Although peer support may benefit both men and women, this evaluation focused on a women-only program designed to address gender-specific barriers to recovery.

Future research could explore the development of comparable peer-support interventions for men, couples, or family-inclusive models, to assess broader applicability. As peer-led models depend heavily on the engagement and well-being of facilitators, future studies should include structured assessments of peer leader satisfaction, role strain, and support needs, to inform long-term program sustainability and scaling. Findings from this feasibility evaluation should be interpreted as exploratory and hypothesis-generating; further research through the ongoing W@H study will provide more robust evidence.

## Conclusions

This feasibility evaluation demonstrated that delivery of the W@H program by trained, volunteer, peer facilitators is feasible and achieved its primary aims of improving coping skills, health empowerment, and health behaviours, and reducing stress and anxiety. Although the W@H program did not include any professional medical or psychological intervention, the group-based, peer-led model provided emotional, informational, and appraisal support. These findings are particularly relevant given that women are less likely to attend CR programs. Peer-support programs, such as W@H, aimed at filling important recovery gaps identified by women with CVD, may be valuable adjuncts to traditional CR programs.
