# Supplementary material for: Peer Support for Women with Heart Disease: Program Description and Evaluation of Women@Heart
Source: CJC Open. 2025 Aug 25;7(12):1638–44. doi: 10.1016/j.cjco.2025.08.008 (PMC12800859; doi:10.1016/j.cjco.2025.08.008)
Supplement: Supplementary Appendix [file mmc1.pdf]

Overview and description of the Women@Heart Sessions

Session	Topic	Description
1	**Women and Heart Disease**	Participants tackle the most common myths and misperceptions of heart disease and gender differences, plus discuss treatments, symptoms, and diagnosis of heart disease.
2	**Road to Recovery**	Participants write their heart disease story and share their personal journey with each other. Participants will also learn about the emotional and physical road to recovery.
3	**Your Emotions and Heart Disease**	Participants explore the most common emotions of surviving a heart incident, including sadness, anger and frustration, in addition to learning the most effective ways of coping with change.
4	**Managing Emotions**	Participants gain specific tools to best manage their emotions, including relaxation techniques, positive thinking, and effective communication strategies.
5	**Manage Your Heart Disease Risk**	Participants learn about goal setting and creating an action plan to manage their risk factors and reduce risk of recurrent events.
6	**Activate Yourself for Health**	Participants receive their personalized Risk Factor Profile, and a road map to identify the health strategies that will have the most positive impact on their health.
7	**Problem Solving Skills**	Participants will discuss the barriers they encounter to making healthy changes and learn to problem-solve challenges and triggers in their everyday lives.
8	**The Slippery Slope of Lifestyle Change**	Participants learn to recognize slips from their health goals and how to build effective strategies to prevent or recover from relapses, including dealing with negative self-talk.
9	**Becoming an empowered patient**	Participants engage in discussion on key tips to improve their risk factors, including healthy eating, physical activity, stress and weight management. Participants also learn about how to remain motivated long term, tackling issues such as confidence, activation and decisional balance.
10	**The Journey goes on…**	Participants look back over the sessions and tie together the most important skills and tools to move confidently into the future.

Summary of open-text responses from Women@Heart participant satisfaction surveys.

1. What did you like about the program?

Key Highlights

- **Supportive Group Atmosphere**
  - Strong appreciation for **peer connection**, **empathy**, and **not feeling alone**.
  - Participants valued a **safe, non-judgmental, and compassionate environment**.
- **Women-Centered Focus**
  - Being in an **all-women group** and discussing **gender-specific issues** (like menopause, women's medications) was consistently praised.
- **Educational Value**
  - The program was seen as very **informative**, offering **practical information** on **heart health, risk factors, and coping** strategies.
  - Participants appreciated learning about **how heart disease affects women** specifically.
- **Small Group Format**
  - **Small groups (around 5)** encouraged participation, sharing, and deeper discussions.
- **Empowerment & Motivation**
  - Many felt **more confident**, **informed**, and **motivated** to take control of their health and advocate for themselves.
- **Program Features & Logistics**
  - The binder and other resources were seen as helpful and practical.
  - Specific praise for:
    - The **Risk Factor Profile** questionnaire
    - The **timing and location** (for some participants)
  - Participants liked that the program helped them **prioritize their health, lower their anxiety**, and develop ways to **manage their condition more effectively**.
  - Many said they were sad to see it end and felt it helped them **start thinking differently about their health**.
- **Key Impact Takeaways**

Participants expressed leaving the program feeling:

- More knowledgeable
- Less isolated
- More in control of their health
- Encouraged by the support of others
- Grateful for a safe, woman-centered environment

2. Do you have suggestions for improvement? – Top Themes

- **Session Format & Duration**
  - Request for **weekly sessions** with a **shorter overall program length** (not 6 months).
  - Many asked for **longer individual sessions** (2.5–3 hours) to allow more sharing time.
- **Group Size & Dynamics**
  - Prefer **smaller, balanced groups** where all voices are heard.
  - Suggested grouping participants by **age or condition** for relevance.
- **Content Delivery**
  - Too much **reading and theory** early on — preference for **more interactive discussions** and **less lecture-style content**.
  - **Goal-setting tools** and **SMART planning** felt overwhelming or unnecessary in group settings.
- **Timing & Location**
  - Start program **sooner after a cardiac event**, ideally **2–4 months post-event**.
  - Several concerns about **venue quality** and accessibility; request for better or additional locations.
- **Expert Involvement & Follow-Up**
  - Strong interest in having **guest speakers (doctors, dietitians)**.
  - Desire for **follow-up sessions** or **online alumni support groups** post-program.

3. Did you find any of the sessions or educational components unhelpful? – Most Common Responses

- **Goal Setting & Worksheets**

    - **SMART goals**, **action planning**, and **in-class worksheets** were frequently seen as confusing, hard to apply, or better suited for **individual sessions**.
- **Too Much Theory**
    - **Content** like **heart anatomy**, **risk factors**, and **types of heart disease** was often seen as **too technical**, **generic**, or **overwhelming**.
    - Some sessions were described as **repetitive** or **overlapping**.
- **Overloaded or Misplaced Content**
    - **Resource session (Session 11)** was valuable but should be **moved to the beginning**.
    - Requests to **tailor content** for specific conditions like **SCAD** and avoid unrelated medical information.
